# Modeling pathways to non‐suicidal self‐injury: The roles of perfectionism, negative affect, rumination, and attention control

**DOI:** 10.1002/jclp.23315

**Published:** 2022-01-20

**Authors:** Kate E. Tonta, Mark Boyes, Joel Howell, Peter McEvoy, Andrew Johnson, Penelope Hasking

**Affiliations:** ^1^ School of Population Health Curtin University Bentley Western Australia Australia; ^2^ Curtin Enable Institute Curtin University Bentley Western Australia Australia; ^3^ Centre for Clinical Interventions Northbridge Western Australia Australia

**Keywords:** attention, emotional cascade, non‐suicidal self‐injury, perfectionism

## Abstract

**Objective:**

Using the Emotional Cascade Model as a theoretical framework, this study tested whether the relationship between perfectionism and non‐suicidal self‐injury (NSSI) operates through rumination and negative affect. Additionally, we tested whether the associations between perfectionism and both rumination and negative affect are moderated by attention control.

**Methods:**

Using a correlational cross‐sectional design, adults aged 18–25 with (*N* = 197) and without (*N* = 271) a history of NSSI completed measures of perfectionism, rumination, negative affect, attention control, and NSSI.

**Results:**

Perfectionism was directly associated with increased odds of NSSI, and indirectly associated with odds of NSSI through rumination and negative affect. The relationship between perfectionism and rumination was moderated by attention focusing, such that the relationship was stronger for individuals who were higher in attention focusing.

**Conclusion:**

Integrating perfectionism and attention with existing models of NSSI may improve understanding of the factors contributing to NSSI and offers insights into future clinical directions.

## 1 INTRODUCTION

Non‐suicidal self‐injury (NSSI) is the deliberate damage to one's body tissue in the absence of suicidal intent (NSSI; International Society for the Study of Self‐Injury, 2018). NSSI can include cutting or scratching the skin, although the range of behaviors is diverse (Swannell et al., 2014). Individuals commonly report engaging in NSSI as a means of regulating particularly intense or unwanted emotions (Taylor et al., 2018). Theoretical models of NSSI, such as the Emotional Cascade Model (Selby et al., 2013), focus on the roles of emotion and rumination in the onset and maintenance of NSSI. Perfectionism is also associated with NSSI, although the nature of this relationship is not clear. Given links between perfectionism and negative emotion (Limburg et al., 2017), rumination (Xie et al., 2019), and biased attention (Tonta et al., 2019), the current study tested whether cascades of negative emotion, driven by poor attention control, may account for the link between perfectionism and NSSI.

### 1.1 Emotional Cascade Model

The Emotional Cascade Model is an emotional regulation model of NSSI and postulates that a positive feedback loop occurs between rumination and negative affect (Selby et al., 2016). Rumination is a cognitive process that involves the repetitive and persistent allocation of attention to one's negative experiences and emotions, and is consistently implicated in the onset and maintenance of adverse psychological outcomes and negative affect (Ehring & Watson, 2008). According to the Emotional Cascade Model, rumination can increase the strength and duration of negative emotions. This relationship is bidirectional and amplifying, where negative affect then increases the degree to which the individual ruminates about their emotional experiences (Selby et al., 2016). When these emotional cascades rapidly intensify, the result is a highly aversive state of distress. With an individual's attention captured by these emotional cascades, individuals who engage in NSSI do so as a “distraction” to interrupt the cascades (Selby & Joiner, 2009). They interrupt these cascades by diverting their attention away from their aversive emotional experience towards elements of the experience of self‐injury such as the pain of injury or the visual stimulus of injury or blood (Selby et al., 2013).

The Emotional Cascade Model, therefore, emphasises the critical role of attention: rumination involves the repeated allocation of attention to negative emotional experiences, and individuals engage in NSSI as a strategy to redirect their attention from and thereby exit emotional cascades. Given the central role of attention, people with greater control over their attention may be less likely to ruminate, and less likely to experience psychological distress. Weaker attention control has been suggested as one mechanism through which rumination is associated with psychological distress (Hsu et al., 2015; Koster et al., 2011).

### 1.2 NSSI and perfectionism

Perfectionism is described as setting self‐worth based on the pursuit of personally demanding high standards, despite adverse consequences (Shafran et al., 2002). One simple example may be where an individual believes they must achieve high grades to be accepted by others. This individual would likely set unachievable high standards for their academic performance (“I must get over 90% in all my assessments”), and this pursuit may come at the cost of other important domains in their life such as interpersonal relationships. Furthermore, the individual may engage in extensive self‐criticism if those standards are not met (e.g., “I am a failure”). Elevated perfectionism is associated with the development and maintenance of a range of adverse psychological outcomes, including depression, anxiety, eating disorders, and obsessive‐compulsive disorders (Limburg et al., 2017), and is consistently correlated with higher negative affect and lower positive affect (Prud'homme et al., 2017; Stoeber et al., 2010; Zuroff et al., 2012).

A systematic review recently summarized the evidence that elevated perfectionism is associated with increased risk of NSSI (Gyori & Balazs, 2021). This association has been demonstrated in adolescents (Luyckx et al., 2015), adults (Claes et al., 2012), and in clinical (Claes et al., 2012) and nonclinical samples (Hoff & Muehlenkamp, 2009). In these cross‐sectional studies, participants with a history of NSSI had significantly higher scores in perfectionism than those with no history of NSSI. However, there is limited evidence for mechanisms that may explain the relationship between perfectionism and NSSI. Investigating this relationship may provide important theoretical information about the mechanisms at play as well as provide new clinical directions for prevention and intervention for NSSI. To make predictions about how perfectionism and NSSI may be related, we must first consider the mechanisms that are known to drive perfectionism.

One transdiagnostic process proposed to explain the relationship between perfectionism and negative affect is rumination. Meta‐analysis has confirmed a robust association between perfectionism and rumination (*r* = 0.20–0.32; Xie et al., 2019). Further, rumination on perfectionistic content explains variance in negative affect above and beyond the variance explained by trait perfectionism (Flett et al., 2002; O'Connor et al., 2007). The idea that individuals with elevated perfectionism ruminate on their perceived failures and flaws may offer an explanation for the common finding that perfectionism acts via rumination to increase negative affect (Xie et al., 2019).

Perfectionism has also been characterized by biased attention to negative information (Howell et al., 2016; Tonta et al., 2019), a pattern that may also contribute to the heightened psychological distress experienced by individuals with elevated perfectionism (Egan et al., 2011). There is evidence demonstrating that individuals with elevated perfectionism preferentially allocate attention to perfectionism‐relevant negative information (e.g., information that signals failure; Howell et al., 2016) and that this bias is characterized by impaired disengagement from negative stimuli (Tonta et al., 2019). It therefore appears that perfectionism, which may be driven by biased attention processes, is associated with some of the key variables in the Emotional Cascades which lead to NSSI.

### 1.3 The current study

The Emotional Cascade Model includes two main components, rumination and negative affect, and points to the critical role of attention allocation. Perfectionism is associated with both these components (rumination and negative affect) of the Emotional Cascade model and may be maintained by biased attention processes. Given the overlap between these concepts of NSSI and perfectionism, two key ideas warrant exploration. First, the relationship between perfectionism and NSSI may be considered in the context of the Emotional Cascade Model such that perfectionism may act as a catalyst for the emotional cascades that lead to NSSI. We, therefore, propose that elevated perfectionism may be associated with greater rumination and negative affect and in turn, increased risk of NSSI. Second, the relationship between perfectionism and emotional cascades may be stronger for individuals with weaker attention control as they are less able to disengage from the emotional cascade than individuals with stronger attention control.

**Figure 1:**
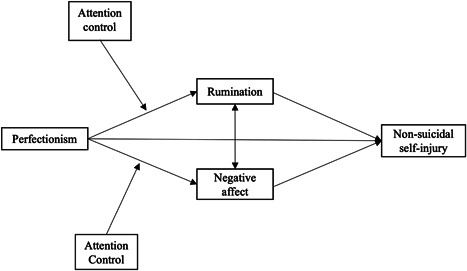
Path analysis model predicting odds of NSSI. NSSI, non‐suicidal self‐injury

In the current study, we test a model that integrates perfectionism, attention control, and NSSI into the framework of the Emotional Cascade Model (Selby & Joiner, 2009) to understand the relationships between these key variables and NSSI. We, therefore, aim to test two hypotheses. First, we hypothesize that perfectionism is associated with NSSI, both directly as well as indirectly, through rumination and negative affect. Second, we hypothesize that the relationships between perfectionism and rumination, and perfectionism and negative affect, are moderated by attention control processes such that weaker attention control is associated with stronger relationships (see Figure 1).

## 2 METHODS

### 2.1 Participants

The study was approved by the Curtin University Human Research Ethics Committee. Data were collected as a part of a broader survey on emotion and cognition. The sample comprised 446 undergraduate university students aged between 18 and 23 years old (*M* = 21.54, SD = 2.44); 110 were male (24.7%) and 336 were female (75.3%). All participants were enrolled at Australian universities, with 354 participants (75.6%) born in Australia, 42 (9.0%) born in South and Southeast Asia, and 19 (4.1%) born in Europe.

### 2.2 Measures

#### 2.2.1 NSSI

The Inventory of Statements About Self‐Injury (ISAS; Klonsky & Glenn, 2009) provides a measure of history, frequency, and type of NSSI behaviors. Participants were presented with a definition of NSSI and reported if they have ever engaged in NSSI (a single item: no/yes). Individuals who indicated they have a history of self‐injury were presented with 12 common methods of NSSI (e.g., cutting, burning) and were asked to estimate a lifetime frequency of each as well as to identify which, if any, was considered to be their main form. Participants were also asked at what age they first engaged in NSSI, and if they have engaged in NSSI in the past 12 months. The ISAS has been used extensively in research with acceptable psychometric properties (4‐week test–retest reliability, *r* = 0.85; 1‐year, *r* = 0.68; Glenn & Klonsky, 2011).

#### 2.2.2 Perfectionism

The Frost Multidimensional Perfectionism Scale—Brief (FMPS‐Brief; Burgess et al., 2016) is an 8‐item measure of perfectionism. In its original form, this measure has two subscales (striving and evaluative concerns). However, emerging research suggests that a bi‐factor model has superior fit to the two‐factor model, and that the general factor accounts for the majority of the shared variance in the bifactor model (Howell et al., 2020). For this reason, the total score was used in this study, which demonstrated strong internal consistency (*α* = 0.86).

#### 2.2.3 Rumination

Rumination was measured using the Repetitive Thinking Questionnaire (McEvoy et al., 2014), which assesses repetitive thinking about one's negative experiences. The RTQ requires individuals to consider how they tend to respond when they feel distressed or upset and then rate how true each of 10 items was of their experience when feeling distressed or upset. The rating scale was a 5‐point scale from 1 (not true at all) to 5 (very true). This measure has demonstrated excellent internal consistency in community samples (*α* = 0.89) and has demonstrated construct validity with measures of negative affect and psychological distress (McEvoy et al., 2010; McEvoy et al., 2014). The internal consistency in the present sample was *α* = 0.94.

#### 2.2.4 Negative affect

Negative affect was measured by the Positive and Negative Affect Schedule (PANAS; Watson et al., 1988). The PANAS is a widely used measure of trait positive and negative affect. The PANAS requires participants to rate to what extent each of the 20 items describe how they generally feel, on a 5‐point scale from 1 (not at all) to 5 (extremely). The measure has two subscales, positive affect (10 items, e.g., enthusiastic) and negative affect (10 items, e.g., distressed); the present study only uses the negative affect subscale. This measure has demonstrated excellent internal consistency in previous research for both the positive (*α* = 0.89) and negative (*α* = 0.85) subscales (Crawford & Henry, 2004). This measure also has demonstrated construct validity with a number of measures of affect, anxiety, and depression (Crawford & Henry, 2004; Watson et al., 1988). The internal consistency in the present sample for negative affect was *α* = 0.90.

#### 2.2.5 Attention control

Attention control was measured using the self‐reported Attentional Control Scale (ACS; Derryberry & Reed, 2002), which evaluates voluntary control over attention and consists of two subscales: attention focusing (7 items) and attention shifting (5 items). Attention focusing refers to the capacity to resist distraction when engaged with stimuli (e.g., “It's very hard for me to concentrate on a difficult task when there are noises around”), while attention shifting refers to the flexibility to move attention away from one stimulus to engage with another (e.g., “It is easy for me to alternate between two tasks”). Participants respond on a 4‐point scale from 1 (almost never) to 4 (almost always), with higher scores representing better attention control. This scale has previously demonstrated strong internal consistency (*α* = 0.82 for Focusing and *α* = 0.71 for Shifting; Judah et al., 2014). Previous research has provided evidence of validity of this measure through significant correlations with other self‐reported measures of attention as well as experimental measures (the anti‐saccade task; Judah et al., 2014). Similarly, internal consistency in the present sample for focusing was *α* = 0.84, and for shifting was *α* = 0.71.

### 2.3 Procedure

Participants responded to online advertisements for undergraduate psychology students participating in research for course credit (*N* = 318). Additional recruitment was conducted via social media advertisements for Australian undergraduate university students interested in participating in research for entry into a prize pool to win an iPad (*N* = 135). Participants provided informed consent at the beginning of the online survey and completion took approximately 60 min for all questions. After completing the measures, participants were provided with resources about self‐injury and stress.

### 2.4 Data analysis plan

Analyses were conducted using Mplus version 8.2 with maximum‐likelihood (ML) estimation and a logit link function for the binary outcome (NSSI no/yes). A path model was tested with one predictor (perfectionism), two mediators (rumination and negative affect), two moderators (attention control—focusing and shifting) on the paths between the predictor and mediators, and one binary outcome (NSSI). The analyses were conducted with bootstrapping with 10,000 samples, and indirect effects were assessed using bias‐corrected bootstrapped confidence intervals (CIs). ML was chosen as it is a “full‐information” procedure that uses all available observations to inform the estimation, compared to weighted least squares (i.e., the Mplus WLSMV estimator), which uses pairwise deletion to handle missing values (Asparouhov & Muthén, 2010). The ML procedure in Mplus also allows for the use of the logit link function, which can be used to produce odds ratios as a measure of effect size. However, using ML with binary variables requires the use of numerical integration, which precludes the calculation of overall model fit statistics (e.g., chi‐square, CFI, TLI; DeMars, 2012). Instead, unstandardized coefficients with 95% CIs are used to assess direct and indirect pathways in the model.

Significant interactions are plotted where the *y*‐axis depicts the relationship between predictor and outcome, and the *x*‐axis is the value of the moderator in units of one standard deviation from the mean. The central line on these graphs depicts the direct effect, while the upper and lower lines represent the upper and lower limits of the 95% CI. Where the lower limit falls below 0 on the *y*‐axis demonstrates where the effect becomes nonsignificant (Bauer & Curran, 2005).

## 3 RESULTS

### 3.1 Preliminary analysis

Of the 446 participants, 190 (41.9%) indicated a history of NSSI. Of those with a lifetime history of NSSI, 104 (54.7%) indicated they had self‐injured in the past 12 months. The three most commonly reported methods of NSSI were cutting (76.3%), banging or hitting oneself (55.3%), and pinching (55.3%). Further, 87 (45.8%) indicated that their main form of self‐injury was cutting, followed by 22 (11.6%) who reported banging or hitting self, and 21 (11.1%) engaged in severe scratching. The mean age of onset of NSSI was 13.6 years (SD = 3.14). Descriptive statistics and correlations between variables in the model are presented in Table 1. The pattern of bivariate correlations was as expected, with NSSI being associated with all variables of interest, as well as gender (more common among women [47.0% of females vs. 23.6% males] *χ* ^2^ = 18.70, *p* < 0.001). Gender was subsequently controlled for in the path model.

**Table 1:** Correlations between variables of interest

	Mean (SD)/*n* (%)		Range	NSSI	Focusing	Shifting	Perfectionism	Negative affect	Rumination
	History of NSSI	No history of NSSI							
NSSI									
Focusing	16.33 (4.32)	18.00 (4.31)	0–21	−0.19**					
Shifting	11.55 (2.62)	12.27 (2.58)	0–15	−0.14*	0.31**				
Perfectionism	27.25 (6.53)	22.81 (6.13)	8–40	0.33**	−0.18**	−0.03			
Negative Affect	29.35 (8.41)	22.83 (7.24)	10–50	0.38**	−0.36**	−0.18**	0.38**		
Rumination	38.76 (8.26)	31.09 (9.13)	10–50	0.39**	−0.40**	−0.17**	0.35**	0.59**	
									
Age	21.73 (2.04)	21.40 (2.67)	18–23	0.07	0.01	−0.01	0.03	−0.05	−0.03
Gender	‐	‐		0.21**	−0.02	−0.09	0.17**	0.18*	0.11*

*Note*: Gender coding (male = 1, female = 2). NSSI coding (no = 0, yes = 1). Correlations including binary variables are point‐biserial.

* *p* < 0.05

** *p* < 0.001.

### 3.2 Tests of direct and indirect effects

**Figure 2:**
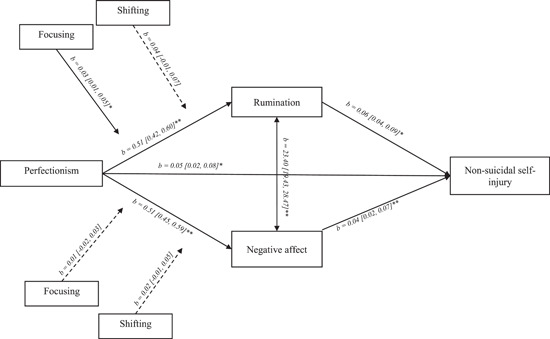
Tested path model with unstandardized b coefficients and associated 95% confidence intervals. Any coefficients with the NSSI as an outcome are in the logit scale. *p < 0.05, **p < 0.001. Dashed lines represent nonsignificant paths. NSSI, non‐suicidal self‐injury

Perfectionism had a direct relationship with odds of NSSI, odds ratio [OR] = 1.05, 95% CI = [1.02, 1.09], *p* = 0.005 (Figure 2). There was no direct relationship between either attention focusing and NSSI (*B* = −0.01 [−0.05, 0.04], *p* = 0.867) or shifting and NSSI (*B* = −0.05 [−0.13, 0.04]*, p* = 0.380). Overall, the model accounted for approximately 29.6% of the variance in NSSI, 26.0% of the variance in rumination, and 26.8% of the variance in negative affect.

### 3.3 Rumination

There was a significant direct relationship between perfectionism and rumination, and a direct relationship between rumination and NSSI. There was also an indirect relationship between perfectionism and odds of NSSI through rumination, OR = 1.03, 95% CI = [1.02, 1.05], *p* < 0.001. Additionally, there were significant direct effects of both focusing (*B* = −0.66 [−0.82, −0.50], *p* < 0.001) and shifting (*B* = −0.36 [−0.64, −0.07], *p* = 0.039) on rumination.

### 3.4 Negative affect

There was a direct effect of perfectionism on negative affect, *B* = 0.51, 95% CI = [0.43, 0.59], *p* < 0.001, and a direct relationship between negative affect and NSSI, OR = 1.04, 95% CI = [1.02, 1.08], *p* = 0.004. There was an indirect relationship between perfectionism and odds of NSSI through negative affect, OR = 1.02, 95% CI = [1.01, 1.04], *p* < 0.001.

There were significant direct effects of both focusing (*B* = −0.45 [−0.58, −0.30], *p* < 0.001) and shifting (*B* = −0.35 [−0.57, −0.12], *p* = 0.028) on negative affect.

## 4 TESTS OF MODERATING EFFECTS

### 4.1 Rumination

**Figure 3:**
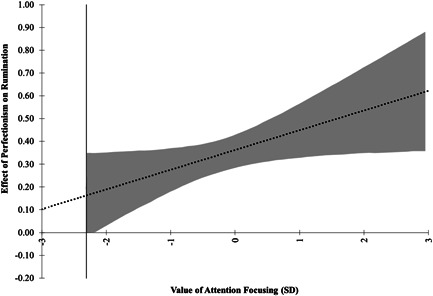
Direct effect of perfectionism on rumination at the mean of attention shifting, moderated by attention focusing (with 95% confidence intervals). Confidence intervals indicated in shading, and vertical marker line indicating bounds of region of significance (from 2.31 standard deviations below the mean)

**Figure 4:**
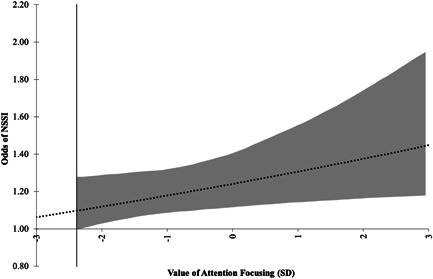
Indirect effect of perfectionism on odds of NSSI via rumination, moderated by attention focusing (with 95% confidence intervals). Confidence intervals indicated in shading, and vertical marker line indicating bounds of region of significance (from 2.38 standard deviations below the mean)

The relationship between perfectionism and rumination was moderated by focusing (*B* = 0.03 [0.01, 0.05], *p* = 0.034; see Figure 3), but not shifting (*B* = 0.04 [−0.00, 0.07], *p* = 0.134; see Figure 4). Interestingly, this relationship was inverse to the direction we hypothesized. Specifically, for individuals scoring more than 2.3 SD below the mean of attention focusing, there was a direct effect of perfectionism on rumination that increased in strength as attention focusing increased (Figure 3).

The indirect effect of perfectionism on odds of NSSI through rumination was stronger among individuals with higher control over their attention focusing. Specifically, there was no indirect effect for individuals scoring 2.3 SD or less below the mean of attention focusing, however, there was an indirect effect for individuals scoring above this threshold (Figure 4).

### 4.2 Negative affect

The relationship between perfectionism and negative affect was not moderated by focusing (*B* = 0.01 [−0.02, 0.03], *p* = 0.644), or shifting (*B* = 0.02 [−0.01, 0.05], *p* = 0.362).

## 5 DISCUSSION

The present study considered the complex relationships between perfectionism, attention control, rumination, and negative affect in relation to NSSI. Adopting the Emotional Cascade model as a theoretical framework, we hypothesized that perfectionism would be associated with NSSI indirectly through both rumination and negative affect. We further hypothesized that the relationships between perfectionism and rumination, and perfectionism and negative affect, would be moderated by attention control processes such that weaker attention control is associated with stronger relationships.

Consistent with previous research, perfectionism was directly associated with NSSI (e.g., Claes et al., 2012; Luyckx et al., 2015), as were rumination and negative affect (e.g., Selby et al., 2013, 2016). As predicted, attention focusing and shifting were also negatively associated with NSSI. Perfectionism was indirectly associated with odds of NSSI through rumination and negative affect, consistent with the hypothesis that perfectionism may predispose the emotional cascades which lead to NSSI.

Although attention focusing and shifting were both negatively associated with NSSI, the relationship between perfectionism and rumination was moderated by attention focusing such that this relationship was stronger for individuals with higher levels of attention focusing. This is the opposite of what we expected. It was expected that when an individual has better attention control, this would be associated with a weaker relationship between perfectionism and rumination as the individual would be more able to volitionally move their attention away from distressing thoughts and feelings. In contrast, better attention control was associated with a *stronger* relationship between perfectionism and rumination. One possible explanation for this finding may be that individuals with elevated perfectionism are at risk of greater rumination and therefore greater risk of NSSI *when* their attention is reallocated preferentially towards perfectionistic stimuli such as perceived failures/flaws. We propose that this may be occurring because individuals with elevated perfectionism use their stronger attention control to intentionally focus on perceived failures or flaws, although further research is needed to test this possibility.

There are a number of possible explanations for our finding that the relationship between perfectionism and negative affect was not moderated by attention. There is a large amount of shared variance between rumination and negative affect, and this overlap may offer one explanation for the nonsignificant finding; see Juarascio et al. (2020) for recent work exploring the overlaps between emotion‐related constructs. Another explanation which may account for this pattern of findings is that attention processes simply do not impact the relationship between perfectionism and negative affect. Previous research has indicated that attention control may drive rumination, and it is *through* rumination that attention control is associated with negative psychological outcomes (Hsu et al., 2015). Therefore, it is possible that attention control does not moderate the relationship between perfectionism and negative affect, but rather exerts influence through its association with rumination.

### 5.1 Theoretical and clinical implications

The findings of this study were consistent with the emotional cascades which may lead to NSSI. Specifically, rumination and negative affect were found to be strongly associated with one another, and there were direct effects from rumination and negative affect to odds of NSSI. These findings also suggest that perfectionism may be considered a risk factor for these emotional cascades, and attention processes may serve to heighten the effects of perfectionism on rumination. This study was an initial test of relationships between attention control and NSSI, demonstrating that as expected, poorer ability to focus one's attention is associated with increased risk of NSSI, as measured by self‐reported attention control.

Perfectionism may therefore be one mechanism through which individuals are at heightened risk of NSSI by facilitating emotional cascades. Given these findings, one way to reduce risk of NSSI may be to reduce perfectionism. Cognitive‐behavioral therapy (CBT) delivered online, face‐to‐face, individually, and in groups is effective for treating perfectionism (Lloyd et al., 2015; Suh et al., 2019), in addition to other related symptoms of psychopathology such as rumination (Cook et al., 2019) and negative affect (Josephine et al., 2017; Linde et al., 2015). It is therefore plausible that these interventions may thus also reduce odds of NSSI. Future research may consider how such interventions impact NSSI.

Another target for intervention, given the current findings, may be changing attention processes, specifically increasing capacity to focus attention, to reduce risk of NSSI. Although for individuals with elevated perfectionism this should be implemented with caution and delivered alongside interventions to ensure that the focus of enhanced attention control is not perfectionistic content. Importantly, despite being statistically significant, the small effect size of the moderating effects of attention on the relationship between perfectionism and rumination is such that there may be little clinical significance with regard to odds of NSSI. Therefore, the direct association between attention control and odds of NSSI may be more clinically relevant. There is a range of techniques across theoretical orientations that may be used to achieve this, but one particularly pertinent example comes from cognitive‐behavioral therapy for perfectionism (Shafran et al., 2018). CBT for perfectionism includes techniques specifically targeted at reducing selective attention by increasing the portfolio of information that an individual bases their self‐esteem upon and increasing flexibility around rules for performance/achievement of goals. In conjunction, several therapeutic techniques such as Socratic dialogues or attention control training may be used to increase attention flexibility and control (Harris & Hayes, 2019; McEvoy, 2019; Rochat et al., 2018). For example, in metacognitive therapy, individuals develop metacognitive awareness, which might help to increase “top down” executive control over attention allocation and facilitate disengagement from unhelpful material (Wells & Papageorgiou, 2003).

### 5.2 Limitations and future research

We used cross‐sectional data as a preliminary test of the relationships between perfectionism, attention processes, and the key variables of the Emotional Cascade Model of NSSI (Selby & Joiner, 2009). The Emotional Cascade Model proposed that associations between rumination, negative affect, and NSSI are a dynamic process, and the current research does not provide information about temporal ordering of these processes. Future work should therefore consider exploring how these relationships may develop and change over time, such as through the use of ecological momentary assessments. Importantly, this model is an application of one theoretical model (the Emotional Cascade Model; Selby & Joiner, 2009), but there are other important theoretical accounts which suggest other processes by which NSSI may regulate affect (e.g., Hasking et al., 2017; Nock, 2010). Future research should explore how perfectionism and attention control may be related to these processes. Additionally, attention control was self‐reported. There are critiques that suggest despite these self‐report measures being directly associated with psychological wellbeing and personality, experimental paradigms may provide a more valid assessment of attention processes (Williams et al., 2017). Our findings may therefore be extended by research using experimental paradigms to measure attention. Furthermore, the measurement of attention control reflects the degree to which an individual perceives their ability to control the allocation of their attention. This does not capture differences in attention processes with respect to emotional valence (positive vs. neutral vs. negative information). Future research may also like to look at how biased attention towards emotionally valenced stimuli may be related. The odds ratio for engagement in NSSI given perfectionism was relatively small (1.05) suggesting that although significant, these findings present a small effect size which must be noted. Finally, although not necessarily a limitation, it is important to bear in mind the nature of the sample in this study; these findings are specific to university students aged 18–25. Future research may consider the pattern of findings in other samples.

## 6 CONCLUSION

The present study provided evidence that perfectionism is associated with NSSI both directly and indirectly through critical components of the emotional cascade model (rumination and negative affect). Our findings suggest that perfectionism may be one factor that increases vulnerability to emotional cascades and, in turn, NSSI and therefore provides an additional potential avenue for intervention. Future prospective and experimental research is required to replicate and extend our findings with longitudinal designs and through the use of experimental measures of attention control and flexibility. We hope this study prompts further research into critically evaluating the precursors to emotional cascades that may lead to NSSI.

## ETHICS STATEMENT

This study was conducted according to the guidelines of the Declaration of Helsinki and approved by the Human Research Ethics Committee of Curtin University (HRE2018‐0536).
